# Neuron Protection by EDTA May Explain the Successful Outcomes of Toxic Metal Chelation Therapy in Neurodegenerative Diseases

**DOI:** 10.3390/biomedicines10102476

**Published:** 2022-10-04

**Authors:** Maria Elena Ferrero

**Affiliations:** Department of Biomedical Sciences for Health, Università degli Studi di Milano, Via Mangiagalli, 31, 20133 Milan, Italy; mariaelena.ferrero@unimi.it

**Keywords:** toxic metals, EDTA chelation therapy, neurodegenerative diseases

## Abstract

Many mechanisms have been related to the etiopathogenesis of neurodegenerative diseases (NDs) such as multiple sclerosis, amyotrophic lateral sclerosis, Parkinson’s disease, and Alzheimer’s disease. In this context, the detrimental role of environmental agents has also been highlighted. Studies focused on the role of toxic metals in the pathogenesis of ND demonstrate the efficacy of treatment with the chelating agent calcium disodium ethylenediaminetetraacetic acid (EDTA) in eliminating toxic metal burden in all ND patients, improving their symptoms. Lead, cadmium, aluminum, nickel, and mercury were the most important toxic metals detected in these patients. Here, I provide an updated review on the damage to neurons promoted by toxic metals and on the impact of EDTA chelation therapy in ND patients, along with the clinical description of a representative case.

## 1. Introduction

Neurodegenerative diseases (NDs) encompass all disorders that affect both the central and peripheral nervous systems and are widespread worldwide. Genetic, congenital, and epigenetic disorders, infections, lifestyle, and exposure to environmental toxins may contribute to the onset of ND. So far, however, since each patient experiences different symptoms, the specific etiopathogenesis has not been fully elucidated, slowing down development of proper, targeted therapies and orientation towards treatments that may fight symptoms. To address this issue, in recent years academics have focused on the role of toxic metal (TM) poisoning in NDs’ progression and described related mechanisms that are directly and indirectly involved in neurotoxicity [1]. In particular, it was found that patients with significant loads of TMs were all affected by ND, and that their severe symptoms were correlated with the severity of intoxication. It was also shown that chelation therapies with ethylenediaminetetraacetic acid (CaNa_2_EDTA) (hereafter EDTA) [1,2,3] are effective in eliminating the burden of TMs in humans, and that EDTA has many other important functions, including antioxidant and anti-inflammatory properties as well as the capacity to protect against endothelial damage [1]. The present review investigates how TMs can be considered among the most important etiological agents in ND, as well as why neuron protection achieved with EDTA may culminate in favorable outcomes for ND patients. 

## 2. Toxic Metals

Heavy metals (HMs) are natural elements whose atomic weight is five times denser than that of water [4]. Due to their multiple applications in the industrial, domestic, agricultural, medical, and technological fields, HMs are widely dispersed in the environment, including the atmosphere, air, and water [5]; as a result, humans are highly exposed to HMs, and we have now learned that human acute and chronic exposure to these metals may elicit systemic toxicity and severe disorders [6,7,8,9,10]. HM exposure essentially involves inhalation, oral, and dermal routes, and their physiological elimination occurs via urine, feces, and hair. Twenty metals have now been identified as being toxic to humans and accordingly classified as toxic metals (TMs). These are aluminum (Al), antimony (Sb), arsenic (As), barium (Ba), beryllium (Be), bismuth (Bi), cadmium (Cd), cesium (Cs), gadolinium (Gd), lead (Pb), mercury (Hg), nickel (Ni), palladium (Pd), platinum (Pt), tellurium (Te), thallium (Tl), thorium (Th), tin (Sn), tungsten (W), and uranium (U). Additionally, copper (Cu), iron (Fe), magnesium (Mg), manganese (Mn), selenium (Se), and zinc (Zn)—so-called essential metals, because they serve physiological roles, participating in various biochemical and physiological processes for cellular viability and tissue development—may exert toxic effects at high concentrations.

Conversely, Cd, chromium (Cr)(VI), Hg, Pb, and As can exert toxic, multi-organ effects at very low, non-threshold concentrations, and have been recently classified as either “known” or “probable” human carcinogens by the United States Environmental Protection Agency (U.S. EPA), and the International Agency for Research on Cancer (IARC) [8].

The most recognized sources of TMs are organophosphorus pesticides, air pollution, smoking, chemotherapeutic drugs, and diagnostic tracers [1]. TM exposure is accompanied by their elevated levels in the blood (acute exposure), from which they move, reaching tissues and organs, where their accumulation can last a long time (chronic exposure) and cause damage. Notably, TMs may cross the blood–brain barrier (BBB) and accumulate in the central nervous system (CNS), which is particularly vulnerable to them [7].

## 3. Toxic Metals as Risk Factors for ND

The role of toxic metals (TMs) as risk factors in the etiopathogenesis of ND has now been recognized, as along with their involvement in inflicting damage to neurons in multiple sclerosis (MS), amyotrophic lateral sclerosis (ALS), Alzheimer’s disease (AD), and Parkinson’s disease (PD). A close association between occupational exposure to metals and pesticides and the spread of ND has been reported [9]. Regarding MS, the role of TMs and of particulates and gaseous pollutants present in the air as putative triggers of both the development and relapse of MS has been shown [10]. Moreover, patients with MS have significantly higher levels of circulating As and Cd compared to controls [11].

With regard to ALS, the role of TMs in ALS susceptibility, onset, and disease progression has been recently discussed [12], and an etiological association between ALS and TMs is clearly supported by the significantly higher levels of TMs in cerebrospinal fluid of patients compared with the reference values [13]. Previous exposure to Hg and/or Pb has also been identified as a risk factor for the onset and progression of ALS [14]. Observational studies further suggest that Pb exposure since childhood may be a possible cause of ALS in adults [15]. Meanwhile, the hypothesis that either consumption of raw fish—often contaminated with Hg—or the use of amalgam for dental procedures could favor the development of ALS has not found experimental evidence so far [16].

Regarding AD, evidence that Al and other metals may cross the blood–brain barrier and accumulate in brain tissues provides a solid rationale for its association with TMs [17,18]. Human epidemiological studies have also shown that Pb and Cd, as well as Mn at high levels, are associated with impaired cognitive function and cognitive decline, suggesting a causal link with AD [19]. Al-driven neurotoxicity may also be considered to be an essential etiological factor in AD [20,21,22,23].

Meanwhile, excessive exposure to both essential and non-essential metals such as Pb, Al, and Hg, which are also contained in pesticides, has been shown to represent a risk factor for PD [24]. Overall, these data may indicate that any single ND expresses differential sensitivity to individual metals, and we now have clear evidence that exposure to a combination of TMs—such as Pb, As, and methyl-Hg (Me-Hg)—may result in a greater impact than the exposure to individual metals in ND [25].

**Figure 1:**
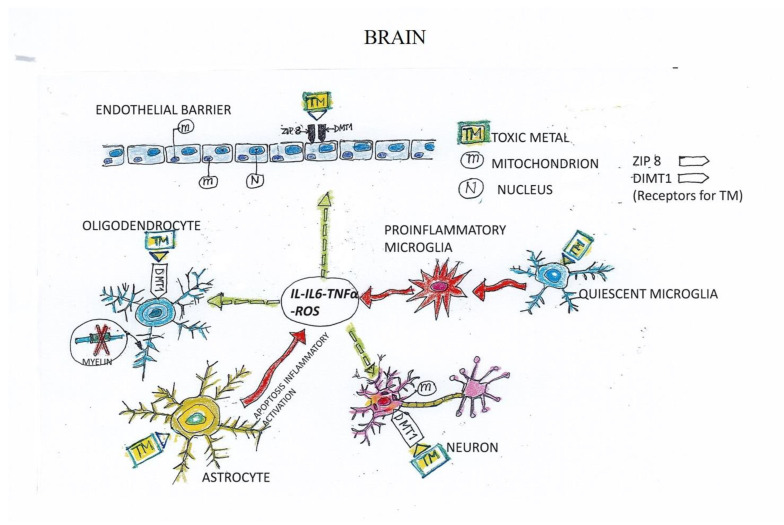
Mechanisms of toxic-metal-inflicted damage on neurological and endothelial cells.

TMs may cross the BBB through trans-/para-endothelial transport and make contact with the cellular components of the brain, where their localization may contribute to the pathogenesis of AD, ALS, or MS ([7] and Figure 1). This mechanism is substantiated by images exhibiting the localization of Hg in the human brain [26], as well as by the presence of very high concentrations of Al in the brains of familial AD patients [22].

TMs cross the endothelial barrier by exploiting the ZIP 8 and DMT1 receptors expressed by the endothelial lining. Then, TMs directly interact with the cellular components of the central nervous system, activating them and inducing a mitochondrial imbalance resulting in increased production of reactive oxygen species (ROS). Quiescent microglia are shifted toward a pro-inflammatory phenotype—characterized by high production of TNF-α, IL-1, and IL-6—that (dashed arrows) indirectly impairs oligodendrocyte myelin production and neuron viability, and promotes endothelial leakage, perpetuating the vicious cycle and the generation of a harmful microenvironment.

In vitro, Pb, Cd, As, and Me-Hg can induce cytotoxic, genotoxic, and apoptotic effects on HT-22 hippocampal cells in a concentration- and potency-dependent manner (Me-Hg > As > Cd > Pb) [27]. Moreover, in vitro co-culturing of oligodendrocytes with neurons has demonstrated that sub-toxic concentrations of HMs may lead to dysfunctional oligodendrocytes, possibly due to imbalanced intracellular Ca^2+^ regulation and altered lipid formation affecting myelin formation [28].

TM-driven CNS toxicity is mainly related to their high concentrations, but gender, genetics, and a prevalent role played by the age of the exposed individuals may also impair their effector function. The latter occurs through mechanisms including mitochondrial dysfunction and compromised DNA repair and neurogenesis, progressively worsening with age [29].

Transport of essential metals into the cells and organelles, where they exert a role as signaling agents or cofactors and, in particular, as activators or redox system components, has been previously reported [30,31]. Several studies indeed demonstrated that reactive oxygen species (ROS) production and oxidative stress play a key role in the toxicity and carcinogenicity of metals such as As [32], Cd [33], Cr [34], Pb [35], and Hg [36]. Alongside oxidative stress, however, the role of Pb-induced epigenetic modifications in eliciting CNS degeneration is becoming increasingly evident [15], and it is plausible that the two mechanisms may coexist. For instance, As causes neurotoxicity, inducing mitochondrial oxidative stress, while Pb directly binds to antioxidants of SH groups of protein cysteine (Cys) [29]. Hg species are transformed through oxidation and methylation before absorption into organisms; Me-Hg forms complexes with Cys and is transported into brain cells via L-type neutral amino-acid transporters; the resulting complex Cys-Me-Hg is responsible for depletion of the antioxidant glutathione (GSH) [29]. In addition, Me-Hg induces mitochondrial ROS and energy failure [36]. Al transport across the BBB can be transferrin-dependent or not. It promotes amyloid beta (Aβ) aggregation and mitochondrial dysfunction, and can also promote oxidative damage through a reaction with H_2_O_2_; it is also able to displace Fe or Ca [29]. Cd toxicity is linked to its ability to bind thiol-containing molecules, impairing cellular redox balance and signaling [29]. Overall, these findings raise the urgent need of maintaining redox homeostasis to limit the oxidative stress in CNS, where neurons and oligodendrocytes are particularly sensitive to ROS and reactive nitrogen species (RNS) [37]. Proteomic analysis has allowed the appreciation of differential protein expression of hippocampal cells associated with the accumulation of Pb, As, and Me-Hg [38]. Since astrocytes are primary homeostatic cells in CNS, the accumulation of TMs in astrocytes may affect some pathways useful in neuroprotection, such as glutamate/GABA glutamine shuttle, antioxidative machinery, and energy metabolism [39]. In AD, Pb, Al, and Cd are involved in the accumulation of toxic proteinaceous species such as β-amyloid and tau proteins [40]. Moreover, As has been shown to affect the hyperphosphorylation and aggregation of tau proteins and may be involved in the deregulation of tau function associated with MS [41].

An interesting hypothesis about the pathogenesis of MS has been formulated, which underlines the role of myelin, involved in the pathway of heme synthesis and, hence, of cytochromes that rely on the heme group [42]. Carbon monoxide and Pb poisoning can indeed cause functional imbalance of the heme group and of heme synthesis, causing myelin damage. Decreased myelin production by oligodendrocytes is a hallmark of MS, making it plausible that TMs can interfere with demyelination [42]. On the other hand, myelination is able to deliver energy via adenosine triphosphate (ATP) to the axons and exert its neurotrophic activity without modifying the mechanism of nerve conduction [43]. Similarly, the mitochondria, which are the main source of ATP, have been identified as a sensitive target of Pb exposure [44].

Blood–brain barrier (BBB) dysfunction provoked by TMs has been suggested by both in vitro and in vivo models of Me-Hg intoxication [45]. In the CNS, the concomitant damage caused by TMs to neurons, as well as to neuron-associated vasculature, could be a pathogenic mechanism of neuroinflammation. Endothelial damage inflicted by TMs has already been reported as a putative cause of the onset of cardiovascular diseases (CVDs). Indeed, Cd, As, and Pb may promote hemorrhagic injury, pathogenic remodeling, and metabolic changes of this complex system, which require interaction of the endothelium with smooth muscle cells as well as the immune and nervous systems [46], and have been shown to be cardiovascular risk factors [47,48]. Moreover, elevated TM levels in the hair of obese patients are associated with cardiovascular complications [49]. Finally, chronic exposure to Hg was associated with an increased risk of fatal/nonfatal ischemic heart disease [50]. The involvement of TMs in inducing endothelial damage and vascular injury responsible for the pathogenesis of both ND and CVD is substantiated by the diagnostic and therapeutic use of cisplatin-based chemotherapy [51] as well as by the use of Gd in magnetic resonance imaging (MRI) to diagnose MS [52].

## 4. Mechanisms of Toxicity Induced by Toxic Metals in ND

TMs cross the cell membrane through the divalent metal transporter 1 (DMT1)—the main transmembrane protein responsible for the uptake of a variety of different cations. For example, uptake of the environmentally relevant hazardous neurotoxic metal Cd in neurons/nerve cells is mediated by overexpression of the membrane metal transporter DMT1 as well as of the Zn transporters ZIP8 and ZIP10 [53]. The involvement of DMT1 in carrying Fe within the brain has recently been highlighted [54,55]. In addition, Al increases DMT1 expression, causing Fe accumulation and alteration of Fe homeostasis in the rat hippocampus [56]. In these conditions, mitochondrial dysfunction occurs, paralleled by the increase in oxidative stress and generation of high levels of mitochondrial ROS [57]. Since mitochondria are essential organelles in the maintenance of neuronal integrity, their damage or dysfunction is associated with neurological disorders. The nervous system is the main target of Pb [58,59], and when oligodendrocytes are affected by Pb, myelin synthesis can be also compromised [42]. The involvement of glial cells other than neurons in neurotoxicity has been reported for Mn [60]. Mn affects mitochondrial function in the glia by producing free radicals and damaging complex II of the electron transport chain, favoring the opening of permeability transition pores in the outer mitochondrial membrane, which allows the release of cytochrome c, triggering caspase-dependent apoptotic pathways [60]. HM,s including Cd, Mn, Hg, As, and Ni, induce impairment of astrocyte functions and direct astrocytic cell death [61]. Activation of microglia upon exposure to Pb has been shown in mice [62]. Moreover, the microglia are able to amplify inflammatory activation of astrocytes in Mn-induced neurotoxicity [63].

Oxidative stress and inflammation are considered to be important pathogenetic pathways in ND. Activated microglia are able to release pro-inflammatory cytokines such as IL-1, IL-6, and TNF-α, as well as ROS [64]. The imbalance of essential metals by toxic metals impairs the structural, regulatory, and catalytic functions of different enzymes, proteins, receptors, and transporters. Neurodegeneration occurs via association of TMs with proteins and subsequent induction of aggregate formation, creating a vicious cycle by disrupting mitochondrial function, which depletes ATP and induces cell death via apoptotic and/or necrotic mechanisms [65].

## 5. Mechanism of TM-Induced Neuron and Endothelial Cell (EC) Damage

Our hypothesis on the mechanisms exploited by toxic metals to damage all cellular components of the CNS is summarized in Figure 1. We propose that TMs in proximity to the endothelial barrier are actively transported into the CNS through the endothelial cell (EC) receptors ZIP8 and DMT1. TMs in the CNS engage the receptor DMT1 expressed by neurons and oligodendrocytes, altering their mitochondrial function, which results in a detrimental overproduction of ROS. Interacting with astrocytes, TMs promote their activation, while when interacting with microglia they promote the shift of quiescent microglia toward a pro-inflammatory phenotype, producing TNF-α, IL-6, and IL-1. The resulting inflamed microenvironment further activates cells in the neighboring tissue and the endothelium, which becomes leaky, perpetuating the vicious cycle. Our hypothesis proposes that damage induced by TMs is ultimately reducible to the generation of severe inflammation, highlighting the critical role exerted by ECs in the onset and progression of ND.

This hypothesis is supported by evidence of TM accumulation in ECs and the ensuing TM-inflicted EC cytotoxicity. For example, Cd induces ZIP8 expression mediated by both NF-κB and JNK signaling [66]. Furthermore, Pb might damage ECs through mitochondrial pathway affecting angiogenesis [67]. Disruption of mitochondrial homeostasis has been associated with premature endothelial senescence and impaired vascular function [68]. High uptake and accumulation into the cytoplasm of inorganic and methyl Hg into the EA.hy926 endothelial cell line have been shown to be correlated with lower Hg efflux [69]. Hexavalent Cr can also damage human umbilical vein ECs through the induction of oxidative stress and activation of P38 MAPK pathways, leading to apoptosis of the cells and CVD [70]. In paraffin-embedded human brain tissue, higher deposits of organic Hg in ECs than in neurons has been shown [71].

## 6. Chelation Therapy with EDTA

Given the mechanism of action promoted by TMs and proposed in Figure 1, therapeutic interventions focused on removing TMs are needed. NDs lack effective treatments or cures and represent major challenges in public health. To alleviate NDs and remove TMs from the blood (accumulation due to acute exposure) or human organs (accumulation due to chronic exposure), a potential strategy may be the binding of TMs with a chelating agent, leading to the formation of a complex that is easier to eliminate. The rationale for the management of EDTA chelation therapy in humans intoxicated by TMs [72] relies on (i) the antioxidant properties expressed by EDTA, (ii) its successful ability to chelate and remove TMs, and (iii) its protective function against cardiovascular events as well as against endothelial activation [1,2,3]. The efficacy of EDTA chelation therapy in removing/lowering the levels of TMs, in eliminating them, and in consistently improving TM-associated symptoms in both ND and CVD—including complications of diabetes—has been widely shown [1,73,74]. The efficacy of EDTA in ND may also depend on its ability to reach the CNS, which has been previously demonstrated by means of biodistribution of labeled EDTA [75]. Meanwhile, the efficacy of EDTA in CVD is supported by the protective effect against renal ischemia induced in a rat model [76].

The routes of administration for chelating agents are oral, intramuscular, and intravenous. TM–chelating agent complexes can be excreted mainly by the kidneys and, to a lesser extent, depending on food intake, by the gastro-enteric tract (i.e., feces) and cutaneous apparatus (i.e., hair and skin annexes). Accordingly, TM levels can be assessed in urine samples collected from patients, following the intravenous “challenge” with the proper chelating agent, used in clinical practice to assess TM contamination [72].

Details about EDTA chelation therapy are provided in the case report below.

## 7. Case Report

We know that MS and most NDs in general are the result of multifactorial agents [77]. To evaluate the role of toxic metals (TMs) in MS and the efficacy of EDTA chelation therapy, we took advantage of two homozygous twin women (IM and EM) characterized by a similar lifestyle, exposure to TMs from adolescence, and identical genetic profile. Both developed MS, at the ages of 15 and 19, respectively. After diagnosis via MRI, both twins were treated with azathioprine. Upon its discontinuation, EM decided to receive immunomodulatory therapy with interferon beta, while IM opted for chelation therapy with the chelating agent EDTA. Their different therapeutic choices allowed us to compare and discuss the differential outcomes. The presence of TMs in the hair and urine of both twins was assessed in our medical center.

MS is a severe neurodegenerative disease. The pathogenic mechanisms involved in MS include both environmental and genetic risk factors. Indeed, the disease is the result of altered homeostasis among genetic predispositions, exposure to pathogens, and pro-inflammatory conditions such as obesity, along with smoking and poor sun exposure/lack of vitamin D [77]. Diagnosis is based on the patient’s clinical history and examination, as well as on multi-organ dissemination (e.g., white and gray matter, brain stem, spinal cord, optic nerve) and spread over time [78].

The use of immunomodulatory/immunosuppressant drugs in the management of MS started many years ago, based on the idea that oligodendrocytes are damaged by leukocytes infiltrating the CNS through the BBB [79,80]. The cancer risk of MS patients undergoing long-term treatment with immunomodulatory/immunosuppressant drugs is still an open and unsolved problem [81]. The long-term use of immunosuppressant drugs has also been associated with several severe side effects in patients undergoing transplantation, as well as those affected by hematological malignancies or autoimmune diseases [82,83].

## 8. Patients

On the basis of the work carried out by both IM and EM, which involved contact with toxic components, we evaluated the presence of TMs in urine and hair, respectively. The protocol was approved by the University of Milan’s Ethics Advisory Committee (number 64/14). All procedures were performed in accordance with the ethical standard of the responsible committee for human experimentation and with the Helsinki Declaration as revised in 2000.

## 9. Chelation Test

This was performed as previously described [2]. Briefly, EDTA (2 g) diluted in 500 mL of physiological saline (Farmax s.r.l., Brescia, Italy) was slowly (over 2 h) administered intravenously to the patient (IM). Urine samples were collected before and for 12 h after the initial intravenous EDTA treatment and analyzed in the Laboratory of Toxicology (Doctor’s Data Inc., St. Charles, IL, USA) through inductively coupled plasma mass spectrometry (ICP-MS). Urine standards—both certified and in-house—were used for quality control and data validation. To avoid a potential error due to fluid intake and sample volume, the results were reported in micrograms (µg) per g of creatinine. Hair samples of EM were evaluated for the presence of TMs using ICP-MS.

## 10. Chelation Therapy and Toxic Metal Analysis

The chelation therapy chosen by IM was performed by a weekly intravenous infusion of 2 g of EDTA in physiological saline for nine months. Further chelation tests were periodically performed to assess the metal burden. The following toxic metals were identified: aluminum (Al), antimony (Sb), arsenic (As), barium (Ba), beryllium (Be), bismuth (Bi), cadmium (Cd), cesium (Cs), gadolinium (Gd), lead (Pb), mercury (Hg), nickel (Ni), palladium (Pd), platinum (Pt), tellurium (Te), thallium (Tl), thorium (Th), tin (Sn), tungsten (W), and uranium (U).

## 11. Clinical Course and Treatments

IM, after a brief period of assembling electronic games with the use of ultrasonic machinery, dedicated herself to the installation of electronic components for television, with a daily commitment of 4 h for four consecutive years (at the age of 14–19 years). At the age of 15, she underwent medical treatment for bilious vomiting, dizziness, and weight loss—all symptoms that forced her to abstain from work for a month. IM experienced tingling on the right side of her body and tetanic contractions, which required hospitalization. Computerized tomography and evoked potentials were performed, and treatment with intravenous corticosteroids was initiated following a diagnosis of labyrinthitis. MS was diagnosed after MRI. IM underwent treatment with oral corticosteroids (25 mg/day) when required. At the age of 19, the patient started attending nursing school.

EM worked for eight hours a day as a tin welder of electronic components, aged between 15 and 18. At the age of 19, she too started nursing school and, concomitantly, showed severely impaired hand movement, which required hospitalization, and was diagnosed with MS following MRI.

The monitoring of disease progression in both patients was carried out using MRI.

**Figure 2:**
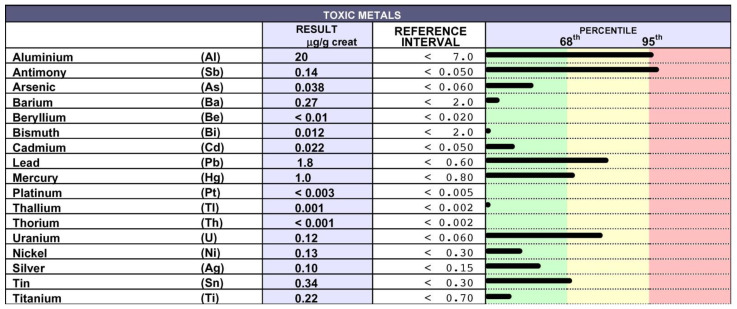
Detection of TMs in the hair of EM: EM did not give consent to the chelation therapy; thus, the presence of TM was detected in her hair using inductively coupled plasma mass spectrometry. Very high/high levels of Al, Sb, Pb, U and, to a lesser extent, Sn and Hg were detected.

The twins received two different drug treatments, and both continued their work as nurses in the hospital. EM was treated with oral corticosteroids following a relapse that occurred when she was 36 years old. She was then treated with azathioprine for a brief period due to poor tolerance, and finally with subcutaneous interferon 1β (Betaferon) for three years, despite the poor tolerance exhibited after a month of treatment, which was mainly associated with head tremors. Despite the toxic metal intoxication index in the hair of EM being very high (Figure 2), she refused the chelation test and, accordingly, chelation therapy.

**Figure 3:**
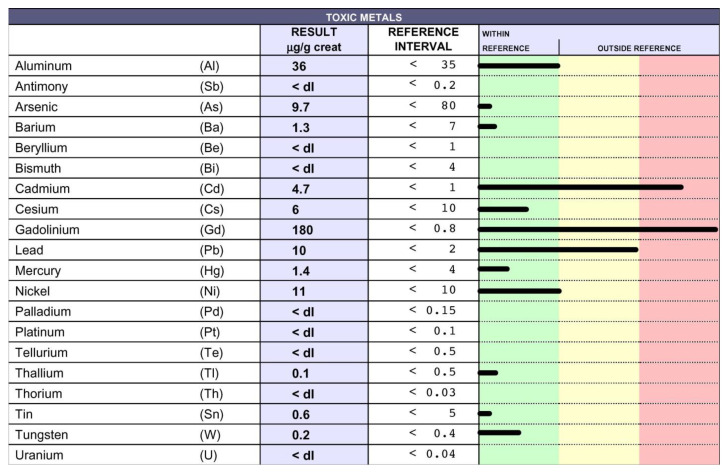
Detection of TMs in the urine samples of IM: IM gave consent to the chelation therapy; thus, the presence of TMs was detected in her urine samples following challenge with EDTA, using inductively coupled plasma mass spectrometry. The chelation test displayed very high levels of Cd, Pb, and Gd.

**Figure 4:**
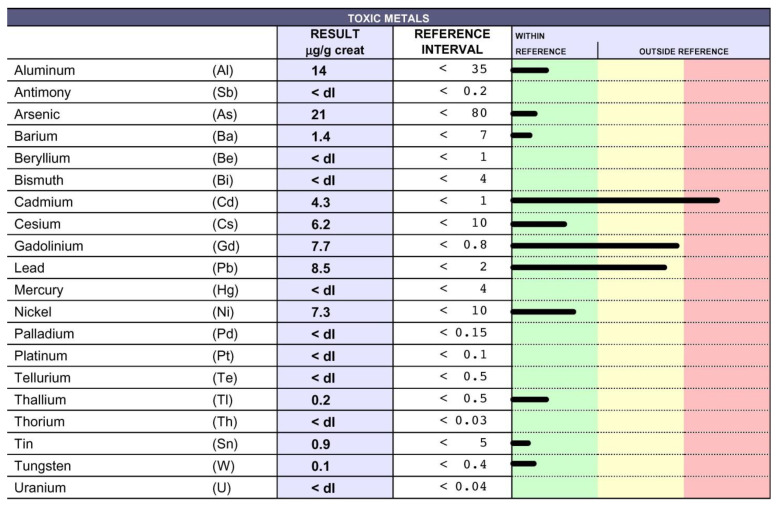
TM levels decreased after EDTA therapy: The presence of TMs in the urine samples of IM following 9 months of therapy with EDTA was evaluated using inductively coupled plasma mass spectrometry. Cd, Pb and, in particular, Gd levels decreased in comparison with those detected before therapy (Figure 3).

IM was treated for five consecutive years (between 19 and 24 years of age) with azathioprine, which was suspended because she was suffering from poor balance and difficulty in moving her right leg. Upon relapse, she was treated with intravenous corticosteroids. At the age of 36, following the challenge with EDTA that detected the presence of TMs in her urine samples (Figure 3), she spontaneously decided to start treatment. After nine months of EDTA chelation treatment (one application per week), the levels of Cd and Pb decreased, and Gd dropped from 180 to 7.7 µg/g (Figure 4). In the Figures the black lines indicate the levels of each toxic metal. Within the green column the values are considered normal, while in the yellow and in the red columns high and very high levels are reported, respectively.

At age of 38 years, both twins underwent percutaneous transluminal angioplasty for the treatment of chronic cerebrospinal venous insufficiency. EM benefited from the surgery in terms of improvement of her quality of life, while IM did not.

Both patients were periodically treated with estroprogestinic hormones because of the presence of ovarian cysts.

At age of 39 years, EM was no longer able to walk, and was therefore bedridden, but chose to continue both interferon-beta and the estroprogestinic treatment. She died of a thromboembolism when she was 40 years old.

IM has continued the EDTA chelation therapies and discontinued the estroprogestinic treatment. She is still alive at the age of 46 and in good health; in particular, she is able to walk without aid and is self-sufficient.

The different outcomes may delineate the possibility that the administration of drugs that orient the immune response could negatively affect fragile, vulnerable subjects, who may benefit from therapies directly targeting the etiological agents, encouraging the management of chelating agents [84].

## 12. Highlights

1. The close association between toxic metals (TMs) and ND definitively points to TMs as relevant etiological agents in ND.
2. We support the hypothesis that the endothelium, whose activation is instrumental to the pathogenesis and spread of ND, could also represent a relevant target in these diseases.
3. Why do only some patients develop ND following exposure to TMs?
  The accumulation of TMs in the cells is dependent not only on their high levels, but also on the capacity of the cells to eliminate them. Successful elimination relies on the physiological, individual sources of antioxidants (e.g., enzymes, vitamins, reduced glutathione, metallothioneins), and functional mitochondria. These form part of the individual assets and may explain the aggravating role of age, which is associated with a biological decline in all of these functions. Consistently, we achieved our best outcomes with chelation therapy with young people, who benefit in terms of improved deambulation, disturbance of fine motor skills, paresthesia and ataxia, and quality of life, substantiating the need for early diagnosis and therapeutic intervention.
4. Many researchers emphasize the importance of using the appropriate chelating agent for each metal. For instance, the iron (Fe) chelator PBT434 also modulates the uptake of Fe^2+^ by human brain microvascular endothelial cells [85]. However, most patients are intoxicated by multiple TMs, making it difficult to apply a tailored therapy. Moreover, in our long-lasting experience, we have learned that therapy with the chelator EDTA has widespread effectiveness in promoting the excretion of all TMs.
5. EDTA therapy is a non-invasive treatment and is not associated with either early or late side effects
6. In our experience, we could not find tight correlation between MRI and the clinical progression of MS [86].
7. Finally, we observed the relevance of the early detection of TM poisoning and the ensuing EDTA chelation in achieving successful clinical outcomes.

## Data Availability

The data will be available from the author.
